# Regioselective chemisorption-induced separate deposition of two types of metal nanoparticles on TiO_2_

**DOI:** 10.1016/j.mex.2018.11.005

**Published:** 2018-11-06

**Authors:** Tomokazu Kiyonaga, Akira Heima

**Affiliations:** Department of Materials System Engineering, National Institute of Technology, Kurume College, 1-1-1 Komorino, Kurume, Fukuoka, 830-8555, Japan

**Keywords:** Regioselective chemisorption-induced separate deposition, Nanocomposite, Separate deposition, Metal complex, Regioselective chemisorption

## Abstract

The discovery of the excellent thermal catalytic activity of Au nanoparticles (NPs) for CO oxidation (Haruta et al., 1987 [1]) triggered intensive research on thermal and visible photo-catalysis based on these NPs (Ref. [2]). Recently, catalysts containing two types of metal NPs loaded onto a TiO_2_ support, i.e., NPs consisting of a separate Au photocatalyst (average size: 13 nm) and another noble metal, were developed as highly efficient visible photocatalysts for several important chemical reactions (Tanaka et al., 2013 [3]). Although the visible photocatalytic activities of Au NPs increase as their particle size decreases (Teranishi et al., 2016 [4]), small Au NPs with a narrow size distribution could not be deposited previously because these NPs underwent dissolution and redeposition (Tanaka et al., 2013 [3]). Additionally, little is known about the mechanism of separate deposition. Herein we report a new method involving the chemisorption and subsequent NaBH_4_ reduction of Au(III) complex ions on TiO_2_–Pt. Our method enables the deposition of small Au NPs with a narrow size distribution (average size: 2.5 nm) on the TiO_2_ surface in TiO_2_–Pt (Au/TiO_2_–Pt). The separate deposition was rationalized in terms of the regioselective chemisorption of Au(III) complex ions on the surface of TiO_2_ by measuring the Au(III) complex ion adsorption.

• The chemisorption and subsequent NaBH_4_ reduction of Au(III) complex ions on TiO_2_-Pt led to the deposition of small Au NPs with a narrow size distribution on the TiO_2_ surface of TiO_2_-Pt. These NPs differ from those obtained by using the existing CPH method, which produced Au NPs with a large particle size and a wide size distribution.
• The separate deposition was rationalized in terms of the regioselective chemisorption of Au(III) complex ions on the TiO_2_ surface, although the mechanism of the CPH method was not disclosed.
• Further application of the regioselective chemisorption-induced separate deposition may enable the development of new catalysts.

## Graphical abstract

**Figure:**
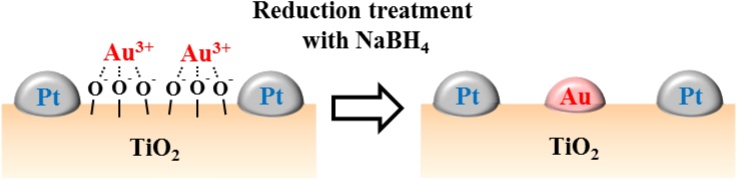

**Specifications Table**

**Table:** 

**Subject Area**	Materials Science
**More specific subject area:**	Materials synthesis and processing
**Method name:**	Regioselective chemisorption-induced separate deposition
**Name and reference of original method**	Colloid photodeposition with a hole scavenger (CPH) method A. Tanaka, K. Nakanishi, R. Hamada, K. Hashimoto, H. Kominami, *ACS Catal.* 3 (2013) 1886-1891.
**Resource availability**	All reagents and solvents (guaranteed reagent) are commercially available and were used as received without further purification.

## Method details

A current topic in the field of catalysis is the discovery that gold, dispersed as nanoparticles (NPs) on metal-oxide supports, such as TiO_2_, exhibits high thermal and visible photo-catalytic activities, even though it is inactive in the bulk state [1,2]. Recently, the separate deposition of Au NPs (average size: 13 nm) and other metal NPs on TiO_2_ was developed as an effective method for enhancing the visible photocatalysis of Au NPs [3]. The visible photocatalytic activity of Au NPs increases as the size of these NPs decreases [4]; however, small Au NPs with a narrow size distribution could not be deposited previously because of the dissolution and redeposition of Au NPs [3]. The new method enabled the separate deposition of small Au and Pt NPs. Furthermore, the origin of the separate deposition is discussed based on the adsorption isotherms of the Au(III) complex ions.

All reagents and solvents (guaranteed reagent) used for the preparation of Au/TiO_2_–Pt are commercially available and were used as received without further purification. Anatase-type TiO_2_ particles (A-100, Ishihara Sangyo) were used to support the noble-metal NPs. In the first step, Pt NPs were loaded onto TiO_2_ (TiO_2_–Pt) by photodeposition [5] using an ultraviolet light-emitting diode (NCSU033B, Nichia). The deposition of Pt NPs on the TiO_2_ particles was carried out by irradiating (*I*_300-390_ = 7.0 mW cm^−2^) a 1.9 mM H_2_PtCl_6_ aqueous solution (50 mL) containing TiO_2_ particles (2 g). After washing and centrifuging the TiO_2_-Pt particles three times, they were dried in vacuum followed by heating at 600 °C for 20 h in an Ar atmosphere. In the second step, Au NPs were loaded onto TiO_2_–Pt by chemisorption [6] followed by the subsequent NaBH_4_ reduction of Au(III) complex ions. TiO_2_–Pt (0.5 g) was added to an aqueous solution of 2.43 mM HAuCl_4_ (5 mL) adjusted to pH 6.0 with NaOH. The suspension was stirred and heated at 70 °C for 1 h. The particles were washed three times with distilled water, and then dried under vacuum. After the particles were re-dispersed in a solution of NaBH_4_ in ethanol (30 mL, 48 mM), the suspension was stirred at 25 °C for 60 min. The resultant particles were washed repeatedly with distilled water, and dried under vacuum (Au/TiO_2_–Pt). The second step was also applied to unmodified TiO_2_ to prepare TiO_2_ loaded with Au NPs (Au/TiO_2_) as a comparative sample.

The deposition states of the NPs were observed by transmission electron microscopy (TEM) at an applied voltage of 200 kV (JEM-2100F, JEOL). Additionally, the amounts of Au and Pt to be loaded were determined as follows. The metal deposits on TiO_2_ were dissolved by treating the particles (50 mg) with aqua regia (24 mL). The concentrations of Au^3+^ and Pt^4+^ ions in the resultant solutions were determined by inductively coupled plasma atomic emission spectroscopy (SPS 3500 DD, Hitachi), and the amounts of Au and Pt to be loaded were calculated as follows;

Loading amount (wt%) = [Concentrations of noble metal ions (mg L^−1^) × 0.024 (L)/50 (mg)] × 100

**Fig. 1:**
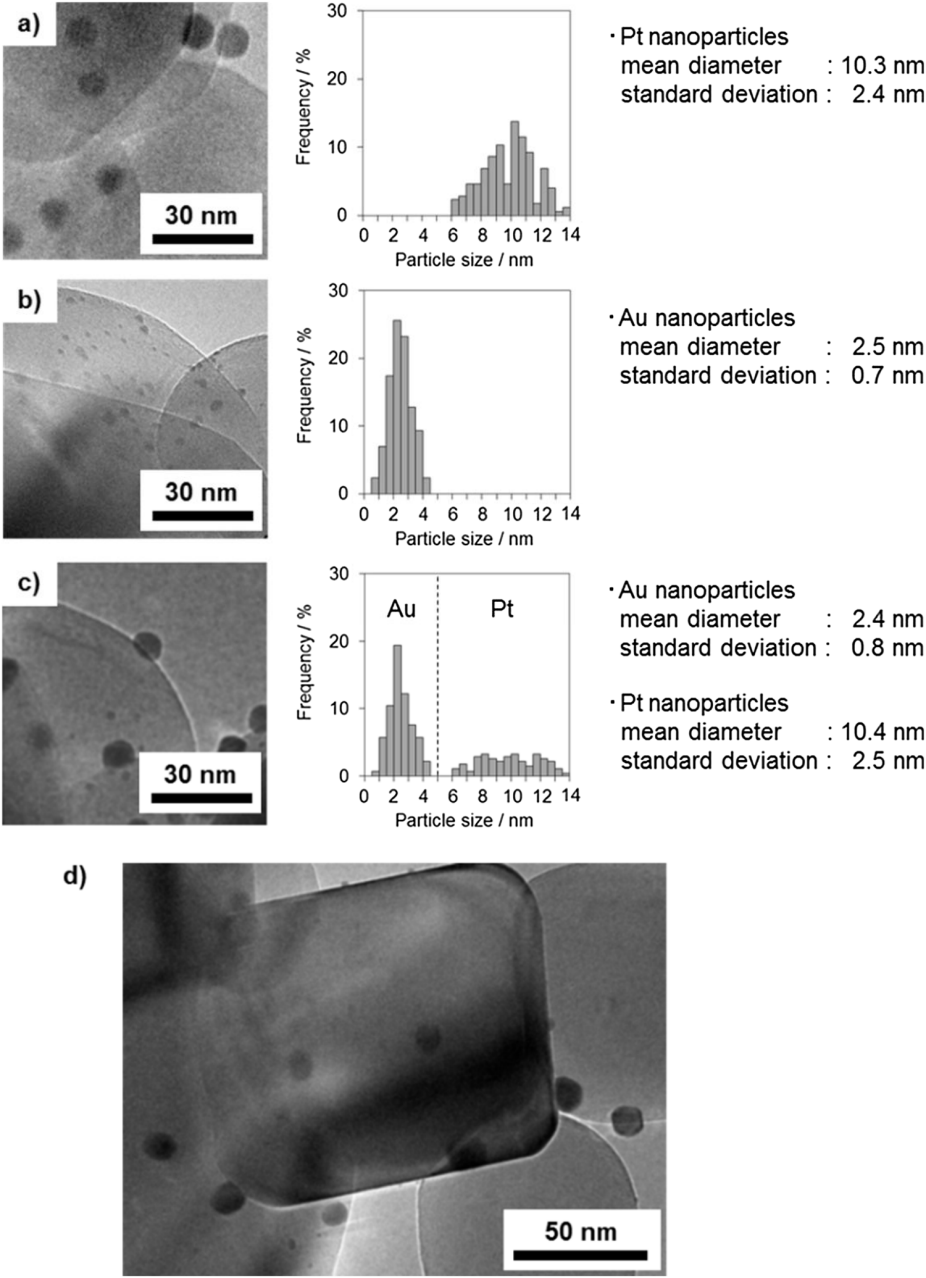
TEM images (left) and size distributions (right) of (a) TiO_2_-Pt (0.20 wt%), (b) Au(0.19 wt%)/TiO_2_, (c) Au(0.18 wt%)/TiO_2_-Pt(0.20 wt%), and (d) Au(0.18 wt%)/TiO_2_-Pt(0.20 wt%) observed at low magnification.

Fig. 1(a) shows a TEM image of TiO_2_–Pt containing 0.20 wt% Pt. Pt NPs with a mean diameter of 10 nm are observed on the TiO_2_ surface. The TEM image of Au(0.19 wt%)/TiO_2_ (Fig. 1(b)) indicates a mean Au NP diameter of 2.5 nm within a relatively narrow size distribution. In the TEM image of Au(0.18 wt%)/TiO_2_–Pt(0.20 wt%) (Fig. 1(c) and (d)), both smaller and larger particles with mean diameters of 2.5 and 10 nm, respectively, are highly dispersed on the TiO_2_ surfaces. On the basis of the TEM images of the Au/TiO_2_ and TiO_2_–Pt particles, these smaller and larger NPs in Au/TiO_2_–Pt were assigned to Au and Pt NPs, respectively. This indicates that the small Au and Pt NPs were individually loaded onto the TiO_2_ surface.

**Fig. 2:**
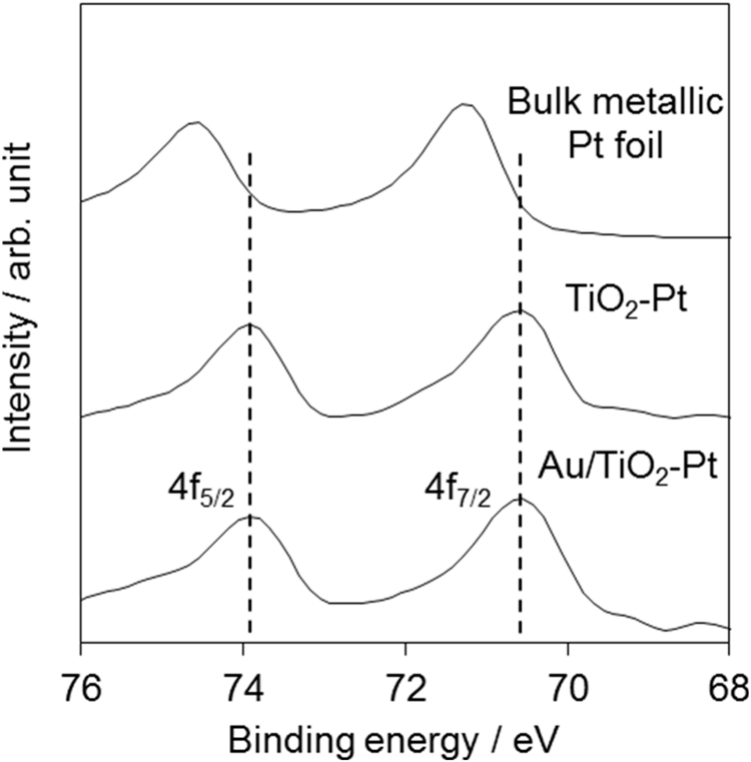
Pt4f XPS spectra of Au/TiO_2_–Pt, TiO_2_–Pt, and bulk metallic Pt foil.

Additionally, to provide strong evidence for the separate deposition of Au and Pt NPs, X-ray photoelectron spectroscopy (XPS; PHI Quantera II, Ulvac-Phi) was carried out using monochromatic Al Kα radiation as the excitation source. The effect of sample charge was reduced by calibrating the XPS binding energies with reference to the C1s peak at 284.8 eV. Fig. 2 shows the Pt4f XPS spectra of Au/TiO_2_–Pt, TiO_2_–Pt, and bulk metallic Pt foil. The Pt 4f_7/2_ and Pt 4f_5/2_ binding energies (*E*_B_) of Au/TiO_2_–Pt (70.6 and 73.9 eV, respectively) agreed well with those of TiO_2_–Pt, although they were lower than those of the bulk metallic Pt foil (71.3 and 74.6 eV, respectively). The spectral differences between TiO_2_–Pt and Pt foil were rationalized in terms of spontaneous electron transfer from TiO_2_ to Pt resulting from the difference in Fermi energy; similarly, the Pt4f binding energy of Au(core)–Pt(shell)/TiO_2_ was reported to be lower than that of TiO_2_–Pt because of the partial electron transfer from Au to Pt [7]. Therefore, the similar binding energies of Au/TiO_2_–Pt and TiO_2_–Pt indicated that the Au and Pt NPs were individually loaded onto the TiO_2_ surface. Thus, a new method for the separate deposition of small Au and Pt NPs on TiO_2_ was established.

**Fig. 3:**
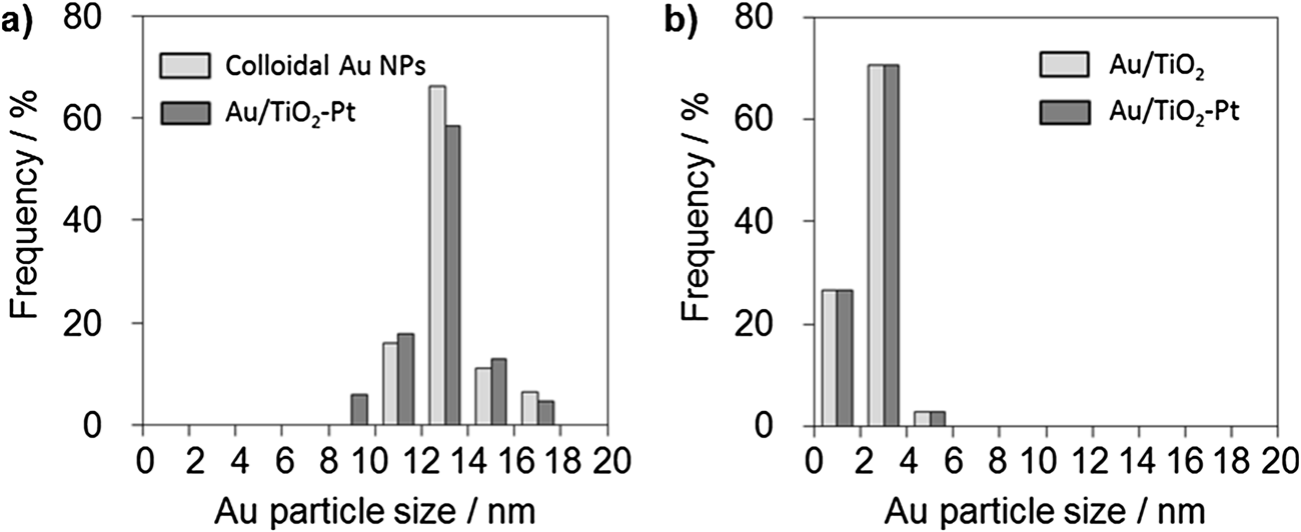
Au particle size distribution in (a) CPH method (Ref. [3a]) and (b) this study.

On the other hand, the colloid photodeposition with a hole scavenger (CPH) method, an existing method for separate deposition, caused the particle size of the deposited colloid Au NPs to increase or decrease probably because of the TiO_2_ photocatalyzed dissolution and redeposition of Au NPs [8] (Fig.3a). Similarly, this phenomenon is involved in the particle size distribution control of small Au NPs in the Au/TiO_2_-Pt. In contrast, the new method provides small Au NPs within a narrow size and similar distribution in Au/TiO_2_-Pt and Au/TiO_2_ (Fig. 3b). The fact that the visible photocatalytic activity of Au NPs increases as the size of these NPs decreases has been reported [4]. Therefore, the deposition of small Au NPs with a narrow size distribution would increase the visible photocatalytic activity. Additionally, the application of the chemisorption of Au(III) complex ions to SrTiO_3_, ZnO, In_2_O_3_, Al_2_O_3_, and so on, has been reported [9]. Therefore, this method will be useful in designing the deposition locations of the small Au NPs and other NPs of various metals such as Pt NPs on various support materials, and the attempt may enable the development of Au photocatalysts. Furthermore, the similar distributions of the Au NPs would be helpful to compare the visible-photocatalytic activities such as those of Au/TiO_2_-Pt and Au/TiO_2_.

**Fig. 4:**
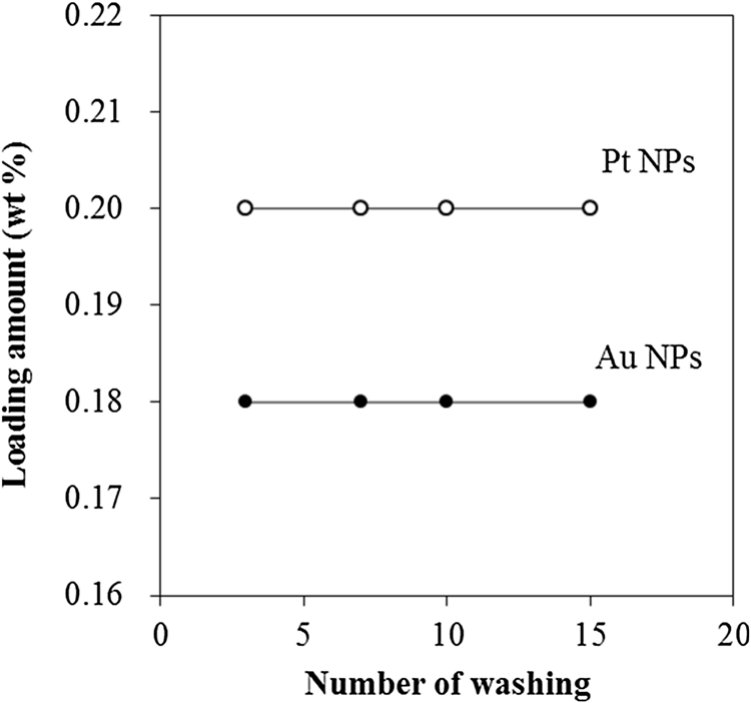
Amounts of loaded Au and Pt NPs during excessive cleaning.

Next, to confirm its stability, Au/TiO_2_–Pt was subjected to excessive cleaning with water (Fig. 4). The amounts of both Au and Pt NPs that were initially loaded remained constant after the cleaning process, verifying the strong connection between the two types of metal NPs (Au and Pt) and TiO_2_. The good stability of Au/TiO_2_–Pt suggests that it can be applied in photocatalysis.

To explain the separate deposition of Au and Pt NPs, the adsorption isotherms of Au(III) complex ions in the samples (TiO_2_ and TiO_2_-Pt) were measured. The samples (0.5 g) were added to an aqueous solution of HAuCl_4_ (5 mL) adjusted to pH 6.0 with NaOH. The suspension was stirred and heated at 70 °C for 1 h for saturated chemisorption [6]. The particles were washed three times with distilled water, and then dried under vacuum. The adsorbed Au(III) complex ions were released by treating the particles (50 mg) with aqua regia (24 mL). The concentrations of Au^3+^ ion in the resultant solutions were determined by inductively coupled plasma atomic emission spectroscopy (SPS 3500 DD, Hitachi), and the amount of Au (III) complex ions that had undergone adsorption was calculated. Under the experimental conditions, the Au(III) complex ions existed primarily as [Au(OH)_3_Cl]^−^ (AuC) [6,10], and the chemisorption of AuC on the surface of TiO_2_ resulted in the formation of a AuC monolayer via the following reaction [6]:

(1) 3Ti_s_OH + [Au(OH)_3_Cl]^−^ → [Au(OTi_s_)_3_] + Cl^−^ + 3H_2_O.

**Fig. 5:**
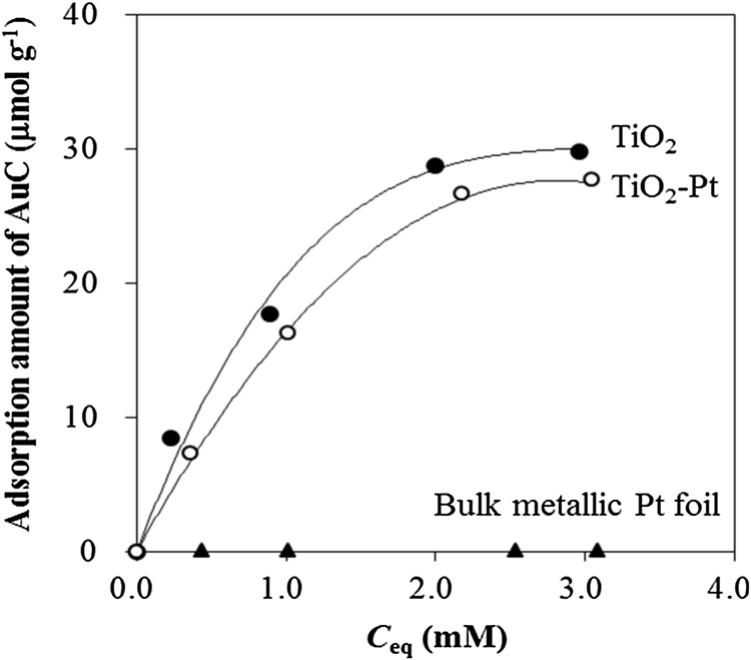
AuC adsorption isotherms on TiO_2_, TiO_2_–Pt, and bulk metallic Pt foil at 70 °C.

Although approximate saturation was reached on TiO_2_ and TiO_2_–Pt at a high concentration of AuC, the amount of AuC adsorbed for TiO_2_–Pt was lower than that for TiO_2_ (Fig. 5). This result could probably be attributed to a decrease in available TiO_2_ surface area upon loading with Pt NPs. Next, the amount of AuC adsorbed for the unsupported Pt NPs was compared with that for TiO_2_. It was difficult to recover the unsupported Pt NPs after the adsorption isotherm experiments; therefore, bulk metallic Pt foil consisting of all Pt planes [11] was used instead of the unsupported Pt NPs, of which the (111) planes are mainly exposed [12]. No adsorption was observed on the surface of the bulk metallic Pt foil, and these results indicate that AuC was regioselectively chemisorbed on the surface of TiO_2_. Additionally, in situ NaBH_4_ reduction of the regioselectively chemisorbed AuC probably led to the separate deposition of Au and Pt NPs. Thus, the chemisorption patterns of AuC revealed a mechanism whereby Au and Pt NPs were separately deposited on the surface of TiO_2_.

This study established a new method for the separate deposition of small Au and Pt NPs on TiO_2_ (Au/TiO_2_–Pt). The regioselective chemisorption of Au(III) complex ions on the surface of TiO_2_ enabled Au and Pt NPs to be deposited separately. This method is expected to be useful for specifying the locations at which the small Au NPs and various other metal NPs are deposited on various support materials. Furthermore, Au and Pd NPs have been separately deposited to develop highly active and selective thermal catalysts based on hydrogen spillover [13], and the thermal catalysis of Au NPs has been shown to reach a maximum at a diameter of approximately 3 nm [2b,2d,9b]. The future application of regioselective chemisorption to accomplish the separate deposition of metal NPs may enable the development of new photocatalysts and thermal catalysts.

## References

[1] Haruta M., Kobayashi T., Sano H., Yamada N. (1987). Chem. Lett..

[2] Haruta M. (2002). CATTECH.

[3] Tanaka A., Nakanishi K., Hamada R., Hashimoto K., Kominami H. (2013). ACS Catal..

[4] Teranishi M., Wada M., Naya S., Tada H. (2016). ChemPhysChem.

[5] Tada H., Takao A., Akita T., Tanaka K. (2006). ChemPhysChem.

[6] Soejima T., Tada H., Kawahara T., Ito S. (2002). Langmuir.

[7] Tada H., Suzuki F., Ito S., Akita T., Tanaka K., Kawahara T., Kobayashi H. (2002). J. Phys. Chem. B.

[8] Kawahara T., Soejima T., Mitsui T., Kiyonaga T., Tada H., Ito S. (2005). J. Colloid Interface Sci..

[9] Naya S., Teranishi M., Kimura K., Tada H. (2011). Chem. Commun..

[10] Murphy P.J., Stevens G., Lagrange M.S. (2000). Geochim. Cosmochim. Acta.

[11] Kim K., Kim K.L., Lee H.B., Shin K.S. (2010). J. Phys. Chem. C.

[12] Shah M.A. (2012). Sci. Iran..

[13] Gu H., Xu X., Chen A., Ao P., Yan X. (2013). Catal. Commun..

